# Oat and Barley in the Food Supply and Use of Beta Glucan Health Claims

**DOI:** 10.3390/nu13082556

**Published:** 2021-07-26

**Authors:** Jaimee Hughes, Sara Grafenauer

**Affiliations:** 1 Grains & Legumes Nutrition Council, 1 Rivett Road, North Ryde, NSW 2113, Australia; j.hughes@glnc.org.au; 2 School of Medicine, University of Wollongong, Northfields Avenue, Wollongong, NSW 2522, Australia

**Keywords:** beta glucan, oats, barley, health claim, regulation, food-health relationship

## Abstract

Beta glucan is a type of soluble dietary fibre found in oats and barley with known cholesterol-lowering benefits. Many countries globally have an approved beta glucan health claim related to lowering blood cholesterol, an important biomarker for cardiovascular disease. However, the use of these claims has not been examined. The aim of this study was to explore the range and variety of oat and barley products in the Australian and global market within a defined range of grain food and beverage categories and examine the frequency of beta glucan health claims. Australian data were collected via a recognised nutrition audit process from the four major Australian supermarkets in metropolitan Sydney (January 2018 and September 2020) and Mintel Global New Product Database was used for global markets where a claim is permitted. Categories included breakfast cereals, bread, savoury biscuits, grain-based muesli bars, flour, noodles/pasta and plant-based milk alternatives and information collected included ingredients lists and nutrition and health claims. Products from Australia (*n* = 2462) and globally (*n* = 44,894) were examined. In Australia, 37 products (1.5%) made use of the beta glucan claim (84% related to oat beta glucan and 16% related to barley beta glucan, specifically BARLEYmax^®^). Of products launched globally, 0.9% (*n* = 403) displayed beta glucan cholesterol-lowering claims. Despite the number of products potentially eligible to make beta glucan claims, their use in Australia and globally is limited. The value of dietary modification in cardiovascular disease treatment and disease progression deserves greater focus, and health claims are an opportunity to assist in communicating the role of food in the management of health and disease. Further assessment of consumer understanding of the available claims would be of value.

## 1. Introduction

Cardiovascular disease (CVD) is a major health problem in Australia, affecting 1.2 million adults [1], yet is largely preventable through the modification of risk factors such as physical inactivity, poor dietary habits and smoking, which together account for up to 90% of the risk of myocardial infarction [2]. Hypercholesterolemia or elevated blood cholesterol is a key risk factor for the development of CVD, and in 2015 accounted for 37% of the burden of coronary heart disease [3]. It is reported that 1.5 million Australian adults had high cholesterol in 2017/18, a condition which often presents with no signs or symptoms. There is a significant opportunity for prevention and treatment of elevated cholesterol and reduction in CVD risk through diet therapy, and one such strategy is focused on adequate consumption of whole grains [4,5]. According to Food Standards Australia New Zealand (FSANZ), whole grain is defined as the intact grain or the dehulled, ground, milled, cracked or flaked grain where the constituents—endosperm, germ and bran—are present in such proportions that represent the typical ratio of those fractions occurring in the whole cereal, and includes wholemeal [6].

Whole grain oats and barley are within the top four largest grain crops in Australia, with 1.6 million tonnes (up 89%) of oats and 12 million tonnes (up 33%) of barley produced in 2020–2021 as a result of a very good growing season [7]. Oats and barley share a favourable, chemically similar polysaccharide beta glucan, (1→3), (1→4)–β–D–glucan and depending on the specific variety, oats contain 6–8% and barley 4–10% *w*/*w* β-glucan [8], which has known cholesterol-lowering properties. The daily consumption of 3 g of beta glucan has been shown to significantly lower blood cholesterol concentrations and reduce circulating low-density lipoprotein cholesterol [9,10,11], key risk factors for CVD. Whole grain oats and barley are the richest sources of beta glucan [8], although specialised plant breeding programs have also produced cultivars with naturally higher levels of beta glucan, such as BARLEYmax^®^, developed by the CSIRO (Commonwealth Scientific and Industrial Research Organisation) [12]. BARLEYmax^®^ is a whole grain barley line that is high in resistant starch and beta glucan (6.4% *w*/*w* beta glucan) and is used as an ingredient in a range of food products in Australia, New Zealand, Japan, USA, Singapore and Malaysia [13].

In Australia and New Zealand, nutrition and health claims are legislated under the Food Standards Code (Standard 1.2.7 Nutrition, Health and Related Claims [14]), which is developed and administered by FSANZ. Currently, there are two existing pre-approved food–health relationships related to beta glucan outlined in Schedule 4 of the Food Standards Code (FSC) [15]. Manufacturers can utilise ‘beta glucan reduces blood cholesterol’, which can be used as the basis for making a high level health claim, or ‘beta glucan reduces dietary and biliary cholesterol absorption’, which can be used as the basis for making a general level health claim [14]. To be eligible, the food product must (1) contain either oat bran, whole grain oats or whole grain barley, (2) provide at least 1 g of beta glucan per manufacturer serving, (3) be accompanied by a dietary context statement (e.g., as part of a balanced diet low in saturated fatty acids and containing 3 g of beta glucan per day) and (4) pass the Nutrient Profiling Scoring Criteria (NPSC), which prevents health claims being carried on products high in energy, saturated fat, sugars and sodium [14,16].

Similar beta glucan claims are approved for use in the United States, Europe, Canada, Brazil, Malaysia, Singapore, Indonesia and South Korea [17]. However, there is no consistency with respect to the prescribed wording of claims or the eligibility criteria between the regulatory bodies [17], except for the universally adopted efficacious daily dose of 3 g beta glucan. The general level and high level health claims on beta glucan and blood lipids, as outlined in the FSC, were based on similar claims that were approved by the Food and Drug Administration and the European Food Safety Authority, respectively.

Although pre-approved beta glucan cholesterol-lowering claims have been permitted in Australia since the introduction of Standard 1.2.7 in 2013, research to date has not examined the frequency of such claims on pack labels. Given the prevalence of hypercholesterolemia globally and in Australia, beta glucan cholesterol-lowering health claims are an opportunity to assist in communicating the role of food in the management of health and disease. Whole grain oats, barley and oat bran have known cholesterol-lowering capabilities, yet the use of these grains in the local and global food supply is unknown. The aim of this study was to explore the range and variety of oat and barley products in the Australian and global market within a defined range of grain food and beverage categories and examine the frequency of beta glucan health claims.

## 2. Materials and Methods

Separate nutrition audits of grain food and beverage categories were conducted from the four major Australian supermarkets (Woolworths, Coles, Aldi, IGA) in metropolitan Sydney and undertaken between January 2018 and September 2020. Approximately 79% of the Australian market share is represented by these supermarkets [18] and were therefore chosen to reflect food choices that the majority of Australians are faced with during food shopping. This recognised process [19] was replicated for each audit category. The food categories included were breakfast cereals (including ready-to-eat cereals, hot cereals, breakfast biscuits, muesli, granola, clusters), bread, savoury biscuits (crackers, crispbread, rice crackers, rice/other grain cakes), grain-based muesli bars, flour, noodles/pasta and plant-based milk alternatives. With permission from store managers, smartphones were used to capture all information on food packaging, including ingredient lists, nutrition panel information and nutrition and health claims. A supplementary internet search was conducted through retailer and identified manufacturer websites to ensure all available products were collected. As a number of products are sold in multiple supermarkets, each product was only recorded once. For products that had multiple pack sizes, the largest pack size was used for data analysis as it was more likely to display a greater number of claims.

As beta glucan health claims are only eligible to be displayed on products made with oat bran, whole grain oats and barley [15], data analysis specifically focused on products containing whole grain oats (e.g., rolled oats, flakes, flour), oat bran, whole grain barley (e.g., barley flour, kibble, puffs) and BARLEYmax^®^. According to FSANZ, a claim is defined as an express or implied statement, representation, design or information in relation to a food or a property of food [20]. For this reason, claims conveyed as text and/or images were included in the analysis. Beta glucan health claims were categorised as either general level or high level as outlined in Standard 1.2.7 of the FSC [14]. General level health claims refer to a nutrient or food component and its influence on general health (e.g., beta glucan reduces dietary and biliary cholesterol absorption), whereas high level health claims are statements that refer to a serious disease or a biomarker of serious disease (e.g., beta glucan reduces blood cholesterol). Compliance of claims according to Standard 1.2.7 was not assessed.

The prevalence of beta glucan heart health/cholesterol-lowering health claims in countries with approved food health relationships was assessed using the Mintel Global New Product Database (Mintel GNPD) [21], an extensive database of food and beverage products launched globally. The number of food and beverage products on the market between January 2018 and September 2020 made with whole grain oats, oat bran, whole grain barley and/or BARLEYmax^®^ were recorded. The time frame and product categories for the Mintel GNPD search were selected to align with the data collection process used for the Australian market. The Mintel GNPD was also used to record the number of products that displayed at least one beta glucan heart health/cholesterol-lowering claim in selected markets conveyed in text or image form. A recent global review of heart health beta glucan claims was used to determine the eligibility for inclusion in the present analysis [17]. According to Mathews et al. (2020), oat beta glucan heart health/cholesterol-lowering claims are permitted in the United States, Europe, Canada, New Zealand, Malaysia, Brazil, Singapore, Indonesia and South Korea [17]. Barley beta glucan claims are permitted in the United States, Europe, Canada and New Zealand. South Korea was excluded from this analysis as the permitted beta glucan claim referred to extracted oat fibre in supplement form, rather than from whole foods [17]. The Mintel GNPD search was conducted on 15 April 2021 using the input parameters outlined in Table 1. Following the search, all products were manually reviewed to ensure that the on-pack beta glucan claims were eligible for inclusion. For non-English food labels, the translated Mintel product description was used to assess on pack claims. Claims not specifically related to oat and/or barley beta glucan and blood cholesterol/heart health were excluded (e.g., whole grain heart health claims permitted in the United States).

### Statistics

Data from photographs taken at each nutrition audit and the Mintel GNPD search output were transcribed into a Microsoft^®^ Excel^®^ spreadsheet (Microsoft 365 MSO Version 16.0.13426.20306, Redmond, WA, USA) for analysis, and checked for errors by an independent reviewer prior to analysis. Descriptive statistics were used to determine the number and proportion of products that contained whole grain oats and/oat bran, whole grain barley and BARLEYmax^®^ and the number and proportion of products that displayed beta glucan heart health/cholesterol-lowering claims within each category.

## 3. Results

Between January 2018 and September 2020, 2462 products from seven food categories were collected from Australian nutrition audits, including 769 breads, 543 breakfast cereals, 363 savoury crackers, 337 noodles/pastas, 173 flours, 165 grain-based muesli bars and 112 plant-based milk alternatives.

### 3.1. Australian Market

A quarter of all food and beverage products were made with whole grain oats and/or oat bran (Table 2), including 78.8% of grain-based muesli bars (*n* = 130), 68% of breakfast cereals (*n* = 369), 9.6% of breads (*n* = 74) and 6.9% of savoury biscuits (*n* = 25). Grain-based muesli bars, breakfast cereals and savoury crackers were made predominantly with rolled oats, while the noodles/pasta products contained added oat fibre.

As shown in Table 2, fewer products contained barley and BARLEYmax^®^ compared to oats (*n* = 195, 14 and 613, respectively). Barley was most commonly found in breads (*n* = 110, 14.3%) as whole grain barley flour or kibbled barley. Seventy-three breakfast cereals (13.4% of subcategory) and a smaller proportion of savoury biscuits, flours, grain-based muesli bars and plant-based milk alternatives contained barley as an ingredient. There were no noodle/pasta products made with barley. BARLEYmax^®^ was found in 14 Australian products overall, most frequently in breakfast cereals, followed by grain-based muesli bars and breads (Table 2).

Table 3 outlines the frequency of beta glucan general and high level health claims in Australia. Across all food and beverage categories examined, only 37 products made a beta glucan health claim (1.5%), and high level health claims were more common than general level health claims. Claims were most often displayed on breakfast cereals (*n* = 32, 5.9% of subcategory). Savoury biscuits, noodles/pasta products and grain-based muesli bars did not display beta glucan health claims. Overall, 84% of all claims were related to oat beta glucan, while the remaining were BARLEYmax^®^ beta glucan claims (16%, *n* = 6). There were no beta glucan claims related to whole grain barley.

### 3.2. Global Market

Of all products launched in the selected markets, 24% contained whole grain oats and/or oat bran (*n* = 10,763), 2.6% contained whole grain barley (*n* = 1188) and nine products contained BARLEYmax^®^ (Table 4). The United States had the highest proportion of oat-containing products relative to all products launched (31.3%, *n* = 1926), followed by New Zealand (24%, *n* = 541), Canada (23.9%, *n* = 679) and Europe (23.7%, *n* = 7068). A small proportion of food and beverage products in Europe were made with whole grain barley (3%, *n* = 940), while less of 2% of products launched in Singapore, Malaysia, New Zealand and Indonesia contained whole grain barley. BARLEYmax^®^ was found in a limited number of products from Malaysia and Singapore only (*n* = 6 and 3 products, respectively).

The initial Mintel GNPD search returned a total of 651 products that made at least one cardiovascular functional claim, including 181 products from the United States, 347 from Europe, 24 from Brazil, 38 from Canada, 26 from Indonesia, 17 from Malaysia, 10 from New Zealand and 8 from Singapore. Following a visual examination of all product labels, 248 products were excluded as the claim did not specifically relate to beta glucan and blood cholesterol. Of note, 52.7% (*n* = 68/129) of the excluded products in the United States made a whole grain and heart health claim (‘Diets rich in whole grain foods and other plant foods and low in total fat, saturated fat and cholesterol may help reduce the risk of heart disease and certain cancers’), which does not relate to beta glucan specifically.

As shown in Table 4, a small proportion of products displayed beta glucan cholesterol-lowering claims on pack labels, indicating that the low usage is not limited to Australia. Europe had by far the greatest number of products that displayed beta glucan claims (*n* = 254), although this only accounted for less than 1% of the total market. In the United States, 52 products made a beta glucan cholesterol-lowering claim, accounting for 0.8% of the US market. Interestingly, the number of products making a whole grain heart health claim, exceeded those making beta glucan claims (*n* = 68 and 52, respectively).

Overall, the majority of claims were made on breakfast cereals (91%, *n* = 365), while a smaller proportion of claims were made on pasta products (*n* = 8), breads (*n* = 7), crackers/biscuits (*n* = 6), wheat- and grain-based snacks (*n* = 5), plant-based milk alternatives (*n* = 3) and cereal bars (*n* = 3), all of which were from the European market.

## 4. Discussion

A range of whole grain oat and barley products are available in the Australian and global market, from intact grains, and as ingredients in a range of food products including breakfast cereals, grain-based muesli bars and breads. However, relative to the number of products that contain whole grain oats, oat bran and barley, the number of claims related to beta glucan appear to be in limited use with <2% of products carrying a claim. The efficacious daily dose of 3 g beta glucan is provided in 75 g of whole grain oats (minimum 5.5% beta glucan) or 55 g of oat bran (4% beta glucan) [22]. For an average adult, this quantity is likely to be difficult to achieve on a regular basis. Whole grain oats, barley and oat bran were the richest sources of beta glucan [8], until the more recent introduction of BARLEYmax^®^, a cultivar with naturally higher levels of beta glucan (6.4% *w*/*w*). BARLEYmax^®^ is 40% higher in beta glucan than rolled oats (personal communications with The Healthy Grain), with 3 g of beta glucan provided within a 45 g serving. Given the smaller volume required to achieve the beta glucan daily target, there is an opportunity for greater use of BARLEYmax^®^ in new product development. It should be noted that work is also underway to develop new wheat lines with higher levels of soluble beta glucan [23].

The data suggests there is limited variety and use of whole grain oats and barley in products other than breakfast cereals and grain-based muesli bars. Previous studies have explored the feasibility of incorporating oats and/or barley into products that require further processing such as bread-making [24] and noodles/pasta [25,26], proving that these whole grains can be utilised as a wheat alternative in a range of food products [27]. This innovation would provide an opportunity to extend the use of oats and barley in products for other meal occasions to assist in achieving the efficacious dose of beta glucan more regularly. Innovative products such as oat rice and oat noodles/pasta that are eligible to carry beta glucan cholesterol-lowering health claims are available in parts of Europe and the Asian market [21], although to our knowledge, such products are not yet commercially available in Australia, presenting an opportunity to expand the use of oats and/or barley in other market sectors. However, it is known that the level of processing of beta glucan is critical for its ability to reduce serum cholesterol levels [28,29,30]. Product innovation should therefore consider use of minimally processed whole grain oats and barley to maintain the food matrix and maximise the beneficial hypocholesteric effects.

Nutrition and health claims have been shown to be helpful in assisting consumers make informed choices and identify healthier food products [31]. Claims also appear to be highly desirable for food industry as evidenced by the increase in product sales following the introduction of a beta glucan heart health claim by Quaker Oats in 1997 [32]. Despite this, the use of beta glucan cholesterol-lowering health claims now appears to be limited, as only 1.5% (*n* = 37) of all Australian products examined carried a claim and just 1–2% of products in other global markets. A possible limitation to the use of beta glucan claims is the serving size required to achieve the beta glucan 1 g dose which may apply to breakfast cereals and the smaller portion grain products (e.g., muesli bars). Furthermore, the additional requirement to pass the NPSC via FSANZ in Australia may place limits on the use of such claims within the snack food category. Greater use of beta glucan health claims may assist in providing consumer awareness of the hypocholesteric benefits of consuming oats and barley, as suggested by Smulders et al. [33] and Ames et al. [27]. Investigation into Australian consumer perceptions of beta glucan cholesterol-lowering claims is an area for future research, using methods utilised in the literature [34,35].

Interestingly, in the United States, the general whole grain heart health claim appeared to be more common than beta glucan related claims although this claim is only available in the US and has been rejected by several other regulatory bodies, FSANZ included. The use of this more general health claim may be more appealing than those relating to beta glucan which is a single food component. The greater use may also relate to perceptions regarding consumer understanding or may simply be aligned with research outcomes for whole grains and CVD. The benefits of regular consumption of whole grain foods are well documented [4,36,37,38,39], with every 16 g increase (one serving) associated with a 9% reduction in cardiovascular disease risk [40,41]. In addition to exploring the Australian consumer understanding of beta glucan claims, a comparison to more general whole grain claims would also be of value especially as whole grain claims are growing rapidly, with a 39% increase in the number of products making whole grain claims in Australia in the past 5 years [21].

Furthermore, a recent cost of illness analysis based on the Australian healthcare system reported the government could save AUD 717.4 million annually in direct and indirect cost for prevention of CVD if all Australian adults (>20 years) consumed the recommended 48 g whole grain daily target intake (DTI) [42]. Despite the profound benefits for individual health and the Australian healthcare system, current consumption of whole grains is poor, at 21 g/day for adults (19–85 years), less than half of the 48 g DTI [43]. As previously suggested, whole grain oats are well placed to form part of nutritious products for the future [44], and the same applies to barley. This presents a significant opportunity for innovation using Australian whole grains to increase intakes in line with dietary recommendations which may have powerful impacts on individual and population health.

To our knowledge, the present study is the first to comprehensively review the use of whole grain oats/oat bran, whole grain barley and BARLEYmax^®^ in the Australian and global food supply, within a defined range of grain food and beverage categories and examine the frequency of beta glucan cholesterol-lowering claims on pack labels. However, some limitations should be noted. While all efforts were made to capture the Australian market in its entirety, differences may exist between geographic areas. Although the MINTEL GNPD reportedly captures 70–80% of all product launches from over 86 economies globally, products may be missed due to the nature of the data collection processes utilised by this organisation. As the beta glucan content of a product is not included in the nutrition information panel unless a nutrition claim is made on the label, eligibility to make beta glucan cholesterol-lowering claims could not assessed.

## 5. Conclusions

Australia is thought to be a world leader in the production of high-quality milling oats and barley for the international market. Given the favourability of growing conditions, there is value in expanding the future application and opportunities for whole grain oats and barley. Despite the sheer number of oat and barley products in the global food market, beta glucan health claims were in limited use and were mostly found on breakfast cereals. Manufacturers could consider selection of oat varieties with higher beta glucan content or enriching products with BARLEYmax^®^ so that products are more likely to be eligible to make beta glucan health claims. Furthermore, the success of beta glucan cholesterol-lowering claims in guiding healthy dietary decisions is dependent on their perception by consumers. Exploring perceptions of beta glucan health claims, and in comparison to whole grain claims, is warranted to gain insight into consumer understanding and value of claims in an Australian context.

## Figures and Tables

**Table 1:** Mintel GNPD search strategy.

Search Variables	Parameters
Market	United States, Europe, Canada, New Zealand, Malaysia, Brazil, Singapore, Indonesia
Date published	Between January 2018 and September 2020
Mintel GNPD categories included	Breakfast cereals; savoury biscuits/crackers; bread and bread products; plant-based drinks; snack/cereal/energy bars; noodles; wheat and other grain-based snacks; pasta
Ingredients included—Oats	Oats and all child ingredients; oat fibre and all child ingredients; oat bran; oat bran flour; oat flour; whole oat flour; oat milk
Ingredients included—Barley	Barley flakes; barley grit; barley groats; barley meal; barley semolina; barley flour; BARLEYmax
Claims	Functional cardiovascular claim

**Table 2:** Number and proportion of food and beverage products made with whole grain oats and/or oat bran, whole grain barely and BARLEYmax^®^, per category.

Food Category	Contains Whole Grain Oats and/or Oat Bran. *n* (% of Total Market)	Contains Whole Grain Barley * n (% of Total Market)	Contains BARLEYmax^®^ *n* (% of Total Market)
Breakfast cereal (*n* = 543)	369 (68.0)	73 (13.4)	7 (1.3)
Bread (*n* = 769)	74 (9.6)	110 (14.3)	3 (0.4)
Savoury biscuits (*n* = 363)	25 (6.9)	3 (0.8)	0
Noodles/pasta (*n* = 337)	9 (2.7)	0	0
Flour (*n* = 173)	3 (1.7)	1 (0.6)	0
Grain-based bars (*n* = 165)	130 (78.8)	5 (3.0)	4 (2.4)
Plant-based milk alternatives (*n* = 112)	3 (2.7)	3 (2.7)	0
Total (*n* = 2462)	613 (24.9)	195 (7.9)	14 (0.6)

* Excluding BARLEYmax^®.^

**Table 3:** Frequency of beta glucan general and high level health claims on products from seven food and beverage categories in Australia.

Food Category	Beta Glucan General Level Health Claim *n* (% of Total Market)	Beta Glucan High Level Health Claim *n* (% of Total Market)
Breakfast cereal (*n* = 543)	0	32 (5.9)
Bread (*n* = 769)	2 (0.3)	0
Savoury crackers (*n* = 363)	0	0
Noodles/pasta (*n* = 337)	0	0
Flour (*n* = 173)	1 (0.6)	0
Grain-based bars (*n* = 165)	0	0
Plant-based milk alternatives (*n* = 112)	0	2 (1.8)
Total (*n* = 2462)	3 (0.1)	34 (1.4)

**Table 4:** Food and beverage products launched between January 2018 and September 2020, containing whole grain oats and/or oat bran, whole grain barley and BARLEYmax^®^ and use of beta glucan heart health/cholesterol-lowering health claims in selected markets.

Market	Contains Whole Grain Oats and/or Oat Bran	Contains Whole Grain Barley	Contains BARLEYmax^®^	Beta Glucan Health Claim
	*n* (% of Total Market)	*n* (% of Total Market)	*n* (% of Total Market)	*n* (% of Total Market)
Europe (*n* = 29,780)	7068 (23.7)	940 (3.2)	0	254 (0.8)
United States (*n* = 6145)	1926 (31.3)	78 (1.3)	0	52 (0.8)
Brazil (*n* = 2991)	493 (16.5)	63 (2.1)	0	17 (0.6)
Canada (*n* = 2838)	679 (23.9)	74 (2.6)	0	31 (1.1)
Indonesia (*n* = 1604)	246 (15.3)	8 (0.5)	0	23 (1.4)
Malaysia (*n* = 552)	116 (21.0)	9 (1.6)	6 (1.1)	10 (1.8)
New Zealand (*n* = 541)	130 (24.0)	8 (1.5)	0	10 (1.8)
Singapore (*n* = 443)	105 (23.7)	8 (1.8)	3 (0.7)	6 (1.4)
Total (*n* = 44,894)	10,763 (24.0)	1188 (2.6)	9 (0.0)	403 (0.9)

## Data Availability

All data for this study are contained within the article.
